# Rapid Diagnosis of Smear-Negative Tuberculosis Using Immunology and Microbiology with Induced Sputum in HIV-Infected and Uninfected Individuals

**DOI:** 10.1371/journal.pone.0001335

**Published:** 2007-12-19

**Authors:** Ronan A. M. Breen, Gareth A. D. Hardy, Felicity M. R. Perrin, Sara Lear, Sabine Kinloch, Colette J. Smith, Ian Cropley, George Janossy, Marc C. I. Lipman

**Affiliations:** 1 Department of Immunology, Royal Free & University College Medical School, London, United Kingdom; 2 Departments of Thoracic and HIV Medicine, Royal Free Hospital London, London, United Kingdom; 3 Department of Primary Care and Population Science, Royal Free & University College Medical School, London, United Kingdom; 4 Centre for Medical Microbiology, Royal Free & University College Medical School, London, United Kingdom; McGill University, Canada

## Abstract

**Rationale and Objectives:**

Blood-based studies have demonstrated the potential of immunological assays to detect tuberculosis. However lung fluid sampling may prove superior as it enables simultaneous microbiological detection of mycobacteria to be performed. Until now this has only been possible using the expensive and invasive technique of broncho-alveolar lavage. We sought to evaluate an immunoassay using non-invasive induced-sputum to diagnose active tuberculosis.

**Methods and Results:**

Prospective cohort study of forty-two spontaneous sputum smear-negative or sputum non-producing adults under investigation for tuberculosis. CD4 lymphocytes specific to purified-protein-derivative of *Mycobacterium tuberculosis* actively synthesising interferon-gamma were measured by flow cytometry and final diagnosis compared to immunoassay using a cut-off of 0.5%. Sixteen subjects (38%) were HIV-infected (median CD4 count [range] = 332 cells/µl [103–748]). Thirty-eight (90%) were BCG-vaccinated. In 27 subjects diagnosed with active tuberculosis, the median [range] percentage of interferon-gamma synthetic CD4+ lymphocytes was 2.77% [0–23.93%] versus 0% [0–2.10%] in 15 negative for active infection (p<0.0001). Sensitivity and specificity of the immunoassay versus final diagnosis of active tuberculosis were 89% (24 of 27) and 80% (12 of 15) respectively. The 3 positive assays in the latter group occurred in subjects diagnosed with quiescent/latent tuberculosis. Assay performance was unaffected by HIV-status, BCG-vaccination or disease site. Combining this approach with traditional microbiological methods increased the diagnostic yield to 93% (25 of 27) alongside acid-fast bacilli smear and 96% (26 of 27) alongside tuberculosis culture.

**Conclusions:**

These data demonstrate for the first time that a rapid immunological assay to diagnose active tuberculosis can be performed successfully in combination with microbiological methods on a single induced-sputum sample.

## Introduction

Prompt detection of tuberculosis (TB) infection is vital for TB control [1]. The development of commercial interferon-gamma (IFN-γ) release assays (IGRA) has focussed attention on the use of host immune reactivity as a marker of infection. Increasing data suggest that blood-based IGRA have significant advantages when investigating latent TB infection (LTBI) compared to the much older immunoassay the tuberculin skin test (TST) [2], [3], [4], [5]. However, both the TST and IGRA are much less helpful in the diagnosis of active TB, especially when this is accompanied by immune suppression such as HIV [6], [7], [8].

Distinct TB antigen-specific responses are found in the lung [9], [10]. Given that TB infection is transmitted typically by inhalation, it is likely that these responses, which are typically of a much higher frequency than found in blood, may be of greater importance than the immunological findings within peripheral blood. Clinical studies using bronchoscopy and broncho-alveolar lavage (BAL) to recover lung cells have confirmed that the pulmonary T-lymphocyte immune response can be used with excellent sensitivity to detect active TB, including non-pulmonary and HIV-related forms [11], [12]. These early data have been confirmed in larger studies of spontaneous sputum-smear negative cases. [13], [14].

Not only is a lung-based approach an exciting advance in TB immune-diagnostics; but also it allows direct smear and culture to be performed on the same primary sample. Given persisting high global rates of drug resistance and the emergence of XDR-TB, the facility to run such immunology and microbiology tests on a single sample is of great potential significance [15].

Obtaining lung fluid by bronchoscopy and BAL is both expensive and invasive [16]. Recently a number of studies have shown that non-invasive sputum induction (SI) using inhaled hypertonic saline for TB diagnosis has a similar microbiological yield to BAL-yet at a substantially reduced cost, and in a significantly more patient-friendly manner [17], [18]. However, cell populations obtained by the two methods differ, which may limit assessment of TB antigen-specific responses when using SI [19].

In this study we have investigated for the first time whether a single sample obtained by simple SI methodology can be used to diagnose TB promptly using immunological assays (flow cytometry and ELISpot) in combination with traditional microbiological techniques.

## Methods

### Subjects

The study was approved by the Royal Free Hospital Ethics Committee (LREC 6236). Adults being investigated for TB who were either (1) sputum acid-fast bacilli (AFB) smear-negative, or (2) not producing sputum and had no positive AFB smears from non-pulmonary samples, were recruited from our urban Teaching Hospital between April 2005 and April 2006. Written informed consent was obtained. Subjects continued to be investigated by their physician independently of SI where appropriate. No subject had commenced anti-tuberculosis medication at the time of study.

SI was performed in a portable, negative-pressure isolation chamber (Elwyn Roberts, Shropshire, UK) using 3% saline delivered via an ultrasonic nebuliser (Sunrise Medical, Wollaston, UK) for 20 minutes. Peak expiratory flow rate (PEFR) was measured every 5 minutes and induction terminated if PEFR declined by >15%, or if chest discomfort were reported. Prior to induction subjects were asked to clear their mouth and nose of any residual saliva or secretions. After each five minute period and prior to any expectoration of sputum this process was repeated. Sputum was expectorated without assistance in to a sterile container.

### Sample handling and laboratory investigation

Sputum was processed within 2 hours. An equivalent volume of 0.1% dithiothreitol solution (Sigma, UK) was added, the sample rolled for 20 minutes at room temperature and an aliquot (usually 50% of the total sample) removed for microbiological investigation.

### Flow cytometry

Absolute leucocyte, lymphocyte and CD4+ T-cell numbers were determined by flow cytometry [20]. Detection of IFN-γ synthesis following 16 hour (overnight) incubation was performed as previously described [11]. In brief, 5×10^4^ CD4 lymphocytes were incubated with tissue culture medium (TCM) alone {negative control}; TCM with PPD (Mycos Research, CO, USA) or TCM with PHA (Sigma) {positive control}. Brefeldin A (Sigma) was added after 2 hours. Harvested cells were fixed and permeabilised, and stained for expression of CD3, CD4 and IFN-γ. Lymphocytes, identified by scatter, were gated according to CD3 expression to produce a histogram of IFN-γ producing CD4+ and CD4- T cells. The IFN-γ+ CD4+ lymphocyte frequency as a percentage of the total CD4+ T-lymphocyte population (%CD4+IFN-γ+) was determined. The %CD4+IFN-γ+ in the negative control was subtracted from that in the PPD-stimulated sample to give a percentage value of PPD-specific CD4+ IFN-γ+ lymphocytes (%PPD-specific CD4+IFN-γ+). No samples were excluded due to high negative control responses which had a median %CD4+IFN-γ+ value of 1.04%. This did not differ significantly between subjects with and without active TB (1.04% versus 0.83% respectively). Using data from early experiments an adequate positive control response was taken as a CD3+ IFN-γ+ response of >10% (median [range] positive control response was 49.3% [16.0–78.3]). All flow cytometry and evaluation of %PPD-specific CD4+IFN-γ+ responses was performed by RAMB.

### ELISpot

ELISpot assays were also performed on induced sputum samples as described previously [21]. Briefly 1×10^5^ leukocytes per well were cultured overnight with duplicates of TCM alone [negative control], PPD or PHA [positive control] in 96 well anti-IFN-γ (Mabtech, Stockholm, Sweden) coated PVDF-backed plates (Millipore, Watford, UK). After overnight incubation IFN-γ spot-forming cells (SFC) were detected according to the manufacturer's instructions (Mabtech). The mean of duplicate conditions was calculated. Results are presented as background subtracted (delta) spot-forming units (ΔSFU) per/10^6^ cells. A negative response to the positive control, PHA, was used to determine assay failure, in which case the result was discounted from the data set. Since none of the PHA treated cells produced a negative result, no assays were excluded. All ELISpot assays and determination of these results were performed by GADH.

### Microbiology

Microscopy acid-fast bacilli (AFB) smears were prepared and stained with auramine-O; and positive smears were confirmed using a Ziehl-Neelsen stain. All positive cultures for *Mycobacterium tuberculosis (M.tb)* were confirmed by the Mycobacterial Reference Laboratory, London, UK.

### Diagnostic definition

Clinical diagnostic decisions were made without reference to immunological data. A final diagnosis of “active TB” was accepted when (1) *M.tb* was cultured or (2) there was clear evidence for a clinical diagnosis based on radiology or histology with an appropriate response to treatment. A final diagnosis of “quiescent or latent TB” was accepted if there were a clearly recorded history of exposure to a smear positive index case with supporting tuberculin skin test (TST) or radiological results but the clinical decision had been made not to commence full anti-tuberculosis therapy. A final diagnosis of “not TB” was accepted if an alternative diagnosis were reached, symptoms resolved rapidly and anti-tuberculosis therapy was not commenced. All subjects without culture-confirmed TB have been followed up for at least 12 months for changes in their diagnostic categorisation.

### Statistical assessment

Comparisons between groups were made using the Wilcoxon test. The data were analysed using SAS version 8.2 (SAS Institute Inc, Cary, NC).

## Results

### Patient information

Forty-two individuals underwent sputum induction. Median age [range] was 32 years [21–63]. Ethnic background was Black 36% (15 of 42), Asian 33% (14 of 42), Caucasian 31% (13 of 42). 90% (38) had received Bacille-Calmette-Guerin (BCG) vaccination. 38% (16 of 42) were HIV-infected with a median [range] blood CD4 count of 332 cells/µl [103–748]. The subjects are described individually in Table 1.

**Table 1:** Description of 42 sputum smear-negative or sputum non-producing subjects undergoing sputum induction for the investigation of possible tuberculosis (subjects are categorised by final diagnoses of active TB; quiescent or latent TB; and not TB, and percentage value of PPD-specific CD4+ IFN-γ+ lymphocytes [%PPD-specific CD4+IFN-γ+])

Country of origin	Age	BCG+	HIV+	Blood CD4 count (cells/ul)	Final diagnosis	AFB smear of IS	IS culture for *Mtb*	%PPD-specific CD4+ IFN-γ+
Angola	34	Yes	Yes	350	Culture-confirmed Pulmonary TB	Negative	Positive	0%
Egypt	29	Yes	No	na	Culture-confirmed Cervical LN TB	Negative	Negative	0%
Pakistan	25	Yes	No	na	Culture-confirmed Testicular and pulmonary TB (no respiratory symptoms)	Positive	Positive	0.13%
Ghana	32	Yes	Yes	350	Culture-confirmed Pulmonary TB	Negative	Positive	0.67%
Bangladesh	25	Yes	No	na	Culture-confirmed Cervical and pulmonary TB (normal CXR)	Negative	Positive	0.67%
Vietnam	32	No	No	na	Culture-confirmed Cervical LN TB	Negative	Negative	0.92%
India	21	Yes	No	na	Culture-confirmed Cervical LN TB	Negative	Negative	1.09%
India	29	Yes	No	na	Presumptive Pulmonary TB	Negative	Negative	1.14%
Somalia	21	Yes	No	na	Culture-confirmed Pulmonary TB	Negative	Positive	1.33%
Somalia	32	Yes	No	na	Presumptive Abdominal TB	Negative	Negative	1.44%
Iran	34	Yes	No	na	Culture-confirmed Pulmonary TB	Negative	Positive	1.81%
Somalia	21	No	No	na	Culture-confirmed Cervical and pulmonary TB (normal CXR)	Negative	Positive	2.09%
Brazil	31	Yes	Yes	748	Culture-confirmed Mediastinal LN TB	Negative	Negative	2.29%
England	29	Yes	No	na	Culture-confirmed Pulmonary TB	Negative	Positive	2.77%
South Africa	32	Yes	Yes	219	Presumptive Pulmonary TB	Negative	Negative	2.96%
Pakistan	24	Yes	No	na	Culture-confirmed Pulmonary TB	Negative	Negative	3.62%
Zimbabwe	54	Yes	No	na	Presumptive Pericardial TB	Negative	Negative	3.75%
Pakistan	31	Yes	No	na	Presumptive Cervical LN TB + erythema nodosum	Negative	Negative	4.17%
Zimbabwe	53	No	Yes	612	Presumptive Mediastinal and abdominal LN TB	Negative	Negative	4.49%
Ireland	36	Yes	Yes	493	Culture-confirmed Pleural TB	Negative	Negative	4.72%
Nepal	27	Yes	No	na	Presumptive Mediastinal LN TB	Negative	Negative	6.98%
Wales	63	Yes	Yes	408	Culture-confirmed Pulmonary TB	Positive	Positive	7.76%
England	55	Yes	No	na	Culture-confirmed Pulmonary TB	Negative	Positive	8.85%
England (Indian descent)	21	Yes	No	na	Culture-confirmed Mediastinal LN TB	Negative	Negative	9.94%
Gambia	23	No	No	na	Culture-confirmed Pulmonary TB	Positive	Positive	15.93%
Bulgaria	33	Yes	No	na	Culture-confirmed Pulmonary TB	Positive	Positive	16.27%
India	28	Yes	No	na	Culture-confirmed Cervical LN TB	Negative	Negative	23.93%
England	61	Yes	No	na	Quiescent/latent TB (TST +. Previous contact of smear+ TB. Concurrent brain tumour)	Negative	Negative	0.72%
Ireland	31	Yes	Yes	459	Quiescent/latent TB (Recent contact of smear+ TB. IDU. Scar on CXR)	Negative	Negative	1.94%
Nigeria	30	Yes	Yes	205	Quiescent/latent TB (Recent contact of smear+ TB)	Negative	Negative	2.10%
DRC	29	Yes	Yes	103	Cough of uncertain cause	Negative	Negative	0%
England (African descent)	30	Yes	No	na	Sarcoidosis	Negative	Negative	0%
India	46	Yes	No	na	LRTI	Negative	Negative	0%
South Africa	27	Yes	No	na	LRTI	Negative	Negative	0.06%
Pakistan	32	Yes	No	na	LRTI	Negative	Negative	0%
Spain	34	Yes	Yes	332	Fever of uncertain cause. Resolved with anti-HIV treatment	Negative	Negative	0.12%
Zimbabwe	36	Yes	Yes	230	Previous MAI	Negative	Negative	0%
England	34	Yes	Yes	303	LRTI	Negative	Negative	0%
China	35	Yes	Yes	313	LRTI	Negative	Negative	0%
Nigeria	53	Yes	Yes	102	Multiple Enlarged LNs. Resolved with anti-HIV therapy	Negative	Negative	0%
Zimbabwe	39	Yes	Yes	150	SLE with cough and fever. Resolved with immunosuppression	Negative	Negative	0.11%
India	31	Yes	No	na	LRTI	Negative	Negative	0%

Abbreviations: AFB = acid-fast bacilli; BCG+ = Bacille-Calmette-Guerin vaccinated; CXR = chest radiograph; DRC = Democratic Republic of Congo; HIV+ = Human immunodeficiency virus positive; IDU = injecting drug user; IS = induced-sputum; LN = lymph node; LRTI = lower respiratory tract infection; Mtb = Mycobacterium tuberculosis; na = not applicable; ND = not done; SLE = systemic lupus erythematosis; TST = tuberculin skin test; TB = tuberculosis

Active TB was diagnosed in 27 subjects of whom 26% (7 of 27) were HIV-infected. 74% (20 of 27) were *M.tb* culture-positive. 56% (15 of 27) had pulmonary and 44% (12 of 27) had active TB diagnosed at a non-pulmonary site.

Three of 42 subjects received a final diagnosis of quiescent or latent TB. A final diagnosis of “not TB” was made in 12 subjects, all of whom had been previously BCG-vaccinated and 7 of 12 (58%) were HIV-infected. These data are shown in Table 2.

**Table 2:** Immunoassay and microbiology results alone and in combination compared to final diagnosis.

	Active TB n = 27	Latent/quiescent TB n = 3	Not TB n = 12	*Active pulmonary TB n = 15*	*Active non-pulmonary TB n = 12*
**HIV+**	7 (26%)	2 (67%)	7 (58%)	4 (27%)	3 (25%)
**BCG+**	23 (85%)	3 (100%)	12 (100%)	13 (87%)	10 (83%)
***Mtb*** ** culture+ from any site**	20 (74%)	0 (0%)	0 (0%)	13 (87%)	7 (58%)
***Mtb*** ** culture+ from IS**	13 (48%)	0 (0%)	0 (0%)	13 (87%)	0 (0%)
**AFB smear+ from IS**	4 (15%)	0 (0%)	0 (0%)	4 (27%)	0 (0%)
**Immunoassay+**	24 (89%)	3 (100%)	0 (0%)	13 (87%)	11 (92%)
**AFB smear or immunoassay+**	25 (93%)	3 (100%)	0 (0%)	14 (93%)	11 (92%)
**IS ** ***Mtb*** ** culture+ or immunoassay+**	26 (96%)	3 (100%)	0 (0%)	15 (100%)	11 (92%)

Subjects diagnosed with active TB are further divided according to whether this was pulmonary or non-pulmonary (italics)

Abbreviations: AFB = acid-fast bacilli; BCG+ = Bacille-Calmette-Guerin vaccinated; HIV+ = HIV-infected; IS = induced-sputum; Mtb = Mycobacterium tuberculosis; TB = tuberculosis

### Induced-sputum cell yields and phenotypes

No induction had to be terminated prematurely and no significant symptoms or reduction in PEFR were noted. The median time after starting induction at which sputum expectoration commenced was 10 minutes [2]–[20] and the median volume expectorated was 5 mls [1]–[20]. The median absolute yield of CD45+ leucocytes was 6.6×10^6^ [1.0–22.5], the percentage of CD45+ leucocytes that were lymphocytes was 4.90% [0.60–41.10%] and the ratio of CD4+ to CD8+ lymphocytes was 1.88 [0.15–29.99].

### PPD-specific IFN-γ response versus final diagnosis

**Figure 1:**
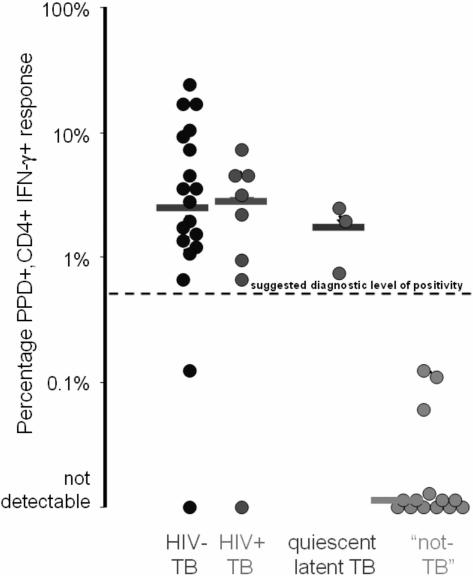
A comparison of final diagnosis against %PPD-specific CD4+IFN-γ+ responses following overnight stimulation of induced-sputum in 42 spontaneous sputum smear negative subjects with possible tuberculosis (medians shown as horizontal bars). The suggested diagnostic cut-off of 0.5% is indicated by the hatched line.

In those for whom the final diagnosis was active TB the median [range] %PPD-specific CD4+IFN-γ+ was 2.77% [0–23.93%]. This was unaffected by HIV co-infection: 2.96% [0–7.76%] for HIV/TB versus 2.43% [0–23.93%] for TB alone (p = 0.87) (Figure 1). In subjects whose final diagnosis was not active TB, the median [range] %PPD-specific CD4+IFN-γ+ was 0% [0–2.10%] (p<0.0001 versus active TB). Delineation of the non-active TB group on the basis of whether the final clinical diagnosis was quiescent/latent TB or not TB revealed %PPD-specific CD4+IFN-γ+ values of 1.94% [0.72%–2.10%] versus 0% [0–0.12%] respectively (Figure 1).

### Induced-sputum as a diagnostic tool–Immunoassay alone and in combination with AFB smear or TB culture

From ROC curve analysis (data not shown), a post-hoc %PPD-specific CD4+IFN-γ+ value of ≥0.5% was selected to define a positive assay. Using this, the assay had a sensitivity of 89% (positive in 24 of 27 of subjects diagnosed with active TB [17 of 20 (85%) with culture-confirmed M.tb and 7 of 7 (100%) who received a presumptive diagnosis]); and a specificity of 80% (negative result in 12 of 15 of subjects where active TB was discounted). All 12 subjects with a final diagnosis of “not TB” had a negative assay.

Amongst those subjects with a final diagnosis of active TB the same induced-sputum sample used for the immunoassay was AFB smear-positive in 15% (4 of 27) of cases; and *M.tb* culture-positive in 48% (13 of 27). It should be noted that culture-confirmation of *M.tb* was obtained in 87% (13 of 15) of pulmonary cases using a single induced-sputum, but only 13% (2 of 15) with spontaneously expectorated sputum in the same subjects. When results were combined using a strategy for prompt diagnosis of AFB smear followed, if negative, by the immunoassay, 93% (25 of 27) had a positive result (4 of 27 [15%] smear-positive and 21 of the remaining 23 [91%] immunoassay-positive). With a strategy of immunoassay and culture, 96% of subjects (26 of 27) were positive (13 [48%] culture-positive, 24 [89%] immunoassay positive and 13 [48%] positive by immunoassay alone). The results according to final clinical diagnosis and site of active TB are displayed in Table 2.

### Induced sputum flow cytometry versus ELISpot

**Figure 2:**
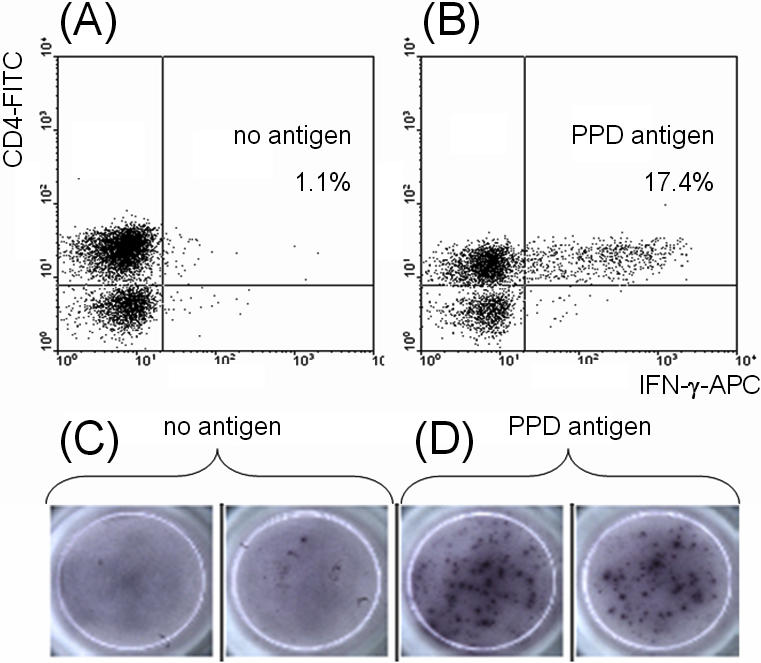
Cytometric dot plots and ELISpot wells from a subject with active tuberculosis, demonstrating interferon (IFN)-γ synthesis in response to overnight incubation of induced-sputum with purified-protein derivative of *Mycobacterium tuberculosis* (PPD). Panel A: Proportion of CD4+ lymphocytes producing IFN-γ after incubation with no antigen (Ag) added. Panel B: Proportion of CD4+ lymphocytes producing IFN-γ after incubation with PPD. Panel C: ELISpot wells showing the IFN-γ T-cell response after incubation with no antigen added. Panel D: ELISpot wells showing the IFN-γ T-cell response after incubation with PPD.

In 9 subjects the IFN-γ response to PPD was measured on the same sample using flow cytometry and ELISpot. In 6 of these the final diagnosis was active TB (3 with pulmonary disease). Equivalent results with both methods were found in all 9 subjects (Table 3; Figure 2).

**Table 3:** Comparison of flow cytometry and ELISpot techniques in assessing PPD-specific interferon-gamma responses

Final diagnosis	Flow cytometry-%PPD-specific CD4+IFN-γ+	ELISpot-IFN-γ SFU/10^6^ cells
Mediastinal and abdominal lymph node TB	4.49%	725
Mediastinal lymph node TB	6.98%	1020
Pulmonary TB	8.85%	725
Pulmonary TB	15.93%	535
Pulmonary TB	16.27%	3405
Cervical lymph node TB	23.93%	4120
Not TB	0%	5
Not TB	0.06%	10
Not TB	0.12%	0

Abbreviations: SFU = spot forming units; TB = tuberculosis;

## Discussion

This is the first study to demonstrate that a single induced-sputum sample can be used for combined immunological and microbiological TB diagnosis. In 42 individuals (38% of whom were HIV-infected) who were either not spontaneously producing sputum or in whom this was AFB smear-negative, our immunoassay had a sensitivity of 89% (24 of 27 positive) and specificity of 80% compared to a final clinical diagnosis of active TB. Although somewhat smaller numbers, these results compare very well with those achieved in blood in HIV-uninfected subjects [7], and ours reported previously using BAL in a similar population [14].

Combining our lung-orientated immunological approach with traditional microbiology produced prompt identification (induced-sputum AFB smear or immunoassay-positive) in 93% of cases; and overall identification (induced-sputum TB culture or immunoassay positive) in 96% of those with active TB at any body site. It should be noted that of the 15 subjects with active pulmonary TB, 4 were AFB smear-positive and 13 were immunoassay-positive on a single induced sputum sample. Thus the latter greatly increased the chance of prompt diagnosis of active, infectious pulmonary TB. The induced sputum immunoassay was also unaffected by HIV status, TB disease site, and BCG vaccination. The similar results obtained using the different methodologies of flow cytometry and ELISpot also demonstrate that the potential of this sputum-based method is not limited to one technique.

Confounding positive responses from BCG-vaccinated individuals without TB have been observed using both the tuberculin skin test (TST) and PPD-based blood assays [22]. This has led to the use of smaller antigens encoded in the region of difference 1 (RD1) portion of the genome of *M.tb,* which is absent in BCG-strains [23]. However, in our lung-based assay, despite all 12 subjects with a clinical diagnosis of “not TB” having received BCG, none had evidence of a detectable pulmonary PPD response. We believe this striking finding reflects the organ-specific pathway taken by vaccination-generated BCG-specific memory lymphocytes ie. trafficking through blood and homing to the skin whilst avoiding the lung [24], [25]. Apart from the insight this offers for future TB vaccination strategies, we feel that these data support the use of PPD, which as a rich antigen mixture, generates higher frequency responses than RD-1 encoded antigens and therefore is likely to be beneficial in the investigation of a condition associated with immune suppression and CD4 lymphopenia in HIV-infected and HIV-uninfected individuals [26], [27], [14].

The sub-optimal specificity observed represents positive assays occurring in 3 subjects with a final diagnosis of quiescent or latent TB. Interestingly these individuals had co-existent conditions which traditionally make TB diagnosis difficult (2 HIV-infection, and 1 terminal malignancy) and reduce the sensitivity of the TST. Given this, it could be argued that a positive result is not such a bad thing as it alerts the clinician *not* to discount TB. That positive results are found in subjects with both quiescent or latent TB and those with active TB confounds all current immune-based assays using interferon-γ expression as the primary readout [28], [29]. However we have previously observed that some of the individuals with these apparent “false-positive” results may develop clearly active TB during follow up [14].

When seeking to delineate between different stages of TB we believe that flow-cytometry adds more value than other immune-based tests, as it can define not only the numbers of cells producing a given cytokine but also the cell phenotype responsible in various states of TB infection. Early studies assessing the expression of both interferon-γ and surface markers of differentiation and memory status such as CD27 have hinted at the promise of such a multi-parameter flow-cytometry based approach [30].

The focus of this study has been the assessment of our induced-sputum assay in diagnosing active TB. The methodology is applicable also to studies of TB pathogenesis. To date, lung-based immunological studies have relied upon bronchoscopy. Although this usefully obtains cell-rich samples, its invasive nature makes it an unpleasant test. Sputum induction offers the possibility of longitudinal assessment of the pulmonary immune response during treatment with good subject acceptability and at greatly reduced cost. We believe that this approach opens up an important area of research which might yield markers of treatment response and even TB cure (31).

These data reveal for the first time how a lung-orientated approach combining a novel immunoassay with traditional microbiology can rapidly diagnose active TB in individuals with smear-negative disease using a single induced-sputum sample. Although a relatively small study performed in a research setting, we believe that our data warrant further work assessing the usefulness of induced-sputum as an alternative to both blood and BAL samples when investigating possible TB.

## References

[1] Keeler E, Perkins MD, Small P, Hanson C, Reed S (2006). Reducing the global burden of tuberculosis: the contribution of improved diagnostics.. Nature.

[2] Brock I, Weldingh K, Lillebaek T, Follmann F, Andersen P (2004). Comparison of Tuberculin Skin Test and New Specific Blood Test in Tuberculosis Contacts.. Am J Resp Crit Care Med.

[3] Ewer K, Deeks J, Alvarez L, Bryant G, Waller S (2003). Comparison of T-cell-based assay with tuberculin skin test for diagnosis of Mycobacterium tuberculosis infection in a school tuberculosis outbreak.. Lancet.

[4] Rangaka MX, Wilkinson KA, Seldon R, Van Cutsem G, Meintjes GA (2007). Effect of HIV-1 infection on T-Cell-based and skin test detection of tuberculosis infection.. Am J Respir Crit Care Med..

[5] Menzies D, Pai M, Comstock G (2007). Meta-analysis: new tests for the diagnosis of latent tuberculosis infection: areas of uncertainty and recommendations for research.. Ann Intern Med..

[6] Dewan PK, Grinsdale J, Kawamura LM (2007). Low sensitivity of a whole-blood interferon-gamma release assay for detection of active tuberculosis.. Clin Infect Dis..

[7] Mazurek GH, Weis SE, Moonan PK, Daley CL, Bernardo J (2007). Prospective comparison of the TST and 2 whole-blood Interferon-γ release assays in persons with suspected tuberculosis.. Clin Infect Dis..

[8] Liebeschuetz S, Bamber S, Ewer K, Deeks J, Pathan AA, Lalvani A (2004). Diagnosis of tuberculosis in South African children with a T-cell-based assay: a prospective cohort study.. Lancet.

[9] Schwander SK, Torres M, Carranza CC, Escobedo D, Tary-Lehmann M (2000). Pulmonary mononuclear cell responses to antigens of Mycobacterium tuberculosis in healthy household contacts of patients with active tuberculosis and healthy.. J Immunol.

[10] Schwander SK, Torres M, Sada E, Carranza C, Ramos E (1998). Enhanced Responses to *Mycobacterium tuberculosis* Antigens by Human Alveolar Lymphocytes during Active Pulmonary Tuberculosis.. J Infect Dis.

[11] Barry SM, Lipman MCI, Bannister B, Johnson MA, Janossy G (2003). Purified protein derivative-activated type 1 cytokine-producing CD4+ T lymphocytes in the lung: a characteristic feature of active pulmonary and non-pulmonary TB.. J Infect Dis.

[12] Breen RAM, Janossy G, Cropley I, Johnson MA, Lipman MCI (2006). Detection of mycobacterial antigen responses in lung but not blood in HIV/tuberculosis co-infected subjects.. AIDS.

[13] Jafari C, Ernst M, Kalsdorf B, Greinert U, Diel R (2006). Rapid Diagnosis of Smear-negative Tuberculosis by Bronchoalveolar Lavage Enzyme-linked Immunospot.. Am J Respir Crit Care Med.

[14] Breen RA, Barry SM, Smith CJ, Shorten RJ, Dilworth JP (2007). The Clinical Application of a Rapid Lung-Orientated TB Immunoassay in Individuals with Possible Tuberculosis.. Thorax [Epub ahead of print].

[15] Raviglione M (2006). XDR-TB: entering the post-antibiotic era?. Int J Tuberc Lung Dis.

[16] McWilliams T, Wells AU, Harrison AC, Lindstrom S, Cameron RJ, Foskin E (2002). Induced sputum and bronchoscopy in the diagnosis of pulmonary tuberculosis.. Thorax.

[17] Conde MB, Soares SL, Mello FC, Rezende VM, Almeida LL (2000). Comparison of sputum induction with fiberoptic bronchoscopy in the diagnosis of tuberculosis: experience at an acquired immune deficiency syndrome reference center in Rio de Janeiro, Brazil.. Am J Respir Crit Care Med.

[18] Brown M, Varia H, Bassett P, Davidson RN, Wall R, Pasvol G (2007). Prospective study of sputum induction, gastric washing, and bronchoalveolar lavage for the diagnosis of pulmonary tuberculosis in patients who are unable to expectorate.. Clin Infect Dis..

[19] Fireman E, Topilsky I, Greif J, Lerman Y, Schwarz Y (1999). Induced sputum compared to bronchoalveolar lavage for evaluating patients with sarcoidosis and non-granulomatous interstitial lung disease.. Respir Med.

[20] Barry SM, Janossy G (2004). Optimal gating strategies for determining bronchoalveolar lavage CD4/CD8 lymphocyte ratios by flow cytometry.. J Immunol Methods.

[21] Lalvani A, Brookes R, Hambleton S, Britton WJ, Hill AV, McMichael AJ (1997). Rapid effector function in CD8+ memory T cells.. J Exp Med..

[22] Pai M, Riley LW, Colford JM (2004). Interferon-gamma assays in the immunodiagnosis of tuberculosis: a systematic review.. Lancet Infect Dis.

[23] Harboe M, Oettinger T, Wiker HG, Rosenkrands I, Andersen P (1996). Evidence for occurrence of the ESAT-6 protein in Mycobacterium tuberculosis and virulent Mycobacterium bovis and for its absence in Mycobacterium bovis BCG.. Infect Immun.

[24] Mora JR, von Andrian UH (2006). T-cell homing specificity and plasticity: new concepts and future challenges.. Trends Immunol.

[25] Hoft DF, Brown RM, Belshe RB (2000). Mucosal Bacille-Calmette-Guerrin vaccination of humans inhibits delayed-type hypersensitivity to purified protein derivative but induces mycobacteria-specific interferon-gamma responses.. Clin Infect Dis.

[26] Zaharatos GJ, Behr MA, Libman MD (2001). Profound T-lymphocytopenia and cryptococcemia in a human immunodeficiency virus-seronegative patient with disseminated tuberculosis.. Clin Infect Dis.

[27] Hirsch CS, Toossi Z, Othieno C, Johnson JL, Schwander SK (1999). Depressed T-cell interferon-gamma responses in pulmonary tuberculosis: analysis of underlying mechanisms and modulation with therapy.. J Infect Dis.

[28] Pai M, Menzies D (2007). Interferon-gamma release assays: what is their role in the diagnosis of active tuberculosis?. Clin Infect Dis..

[29] Kang YA, Lee HW, Hwang SS, Um SW, Han SK, Shim YS, Yim JJ (2007). Usefulness of whole-blood interferon-gamma assay and interferon-gamma enzyme-linked immunospot assay in the diagnosis of active pulmonary tuberculosis.. Chest..

[30] Streitz M, Tesfa L, Yildirim V, Yahyazadeh A, Ulrichs T (2007). Loss of receptor on tuberculin-reactive T-cells marks active pulmonary tuberculosis.. PLoS ONE.

[31] Perrin FM, Lipman MC, McHugh TD, Gillespie SH (2007). Biomarkers of treatment response in clinical trials of novel antituberculosis agents.. Lancet Infect Dis..

